# Endothelial-to-Mesenchymal Transition in Cardiovascular Pathophysiology

**DOI:** 10.3390/ijms25116180

**Published:** 2024-06-04

**Authors:** Aman Singh, Kriti S. Bhatt, Hien C. Nguyen, Jefferson C. Frisbee, Krishna K. Singh

**Affiliations:** 1 Department of Medical Biophysics, Schulich School of Medicine and Dentistry, University of Western Ontario, London, ON N6A 5C1, Canada; asing945@uwo.ca (A.S.); kbhatt23@uwo.ca (K.S.B.); hnguy29@uwo.ca (H.C.N.); jfrisbee@uwo.ca (J.C.F.); 2 Department of Anatomy and Cell Biology, Schulich School of Medicine and Dentistry, University of Western Ontario, London, ON N6A 5C1, Canada

**Keywords:** endothelial-to-mesenchymal transition, EndMT, mechanisms, cardiovascular diseases

## Abstract

Under different pathophysiological conditions, endothelial cells lose endothelial phenotype and gain mesenchymal cell-like phenotype via a process known as endothelial-to-mesenchymal transition (EndMT). At the molecular level, endothelial cells lose the expression of endothelial cell-specific markers such as CD31/platelet-endothelial cell adhesion molecule, von Willebrand factor, and vascular-endothelial cadherin and gain the expression of mesenchymal cell markers such as α-smooth muscle actin, N-cadherin, vimentin, fibroblast specific protein-1, and collagens. EndMT is induced by numerous different pathways triggered and modulated by multiple different and often redundant mechanisms in a context-dependent manner depending on the pathophysiological status of the cell. EndMT plays an essential role in embryonic development, particularly in atrioventricular valve development; however, EndMT is also implicated in the pathogenesis of several genetically determined and acquired diseases, including malignant, cardiovascular, inflammatory, and fibrotic disorders. Among cardiovascular diseases, aberrant EndMT is reported in atherosclerosis, pulmonary hypertension, valvular disease, fibroelastosis, and cardiac fibrosis. Accordingly, understanding the mechanisms behind the cause and/or effect of EndMT to eventually target EndMT appears to be a promising strategy for treating aberrant EndMT-associated diseases. However, this approach is limited by a lack of precise functional and molecular pathways, causes and/or effects, and a lack of robust animal models and human data about EndMT in different diseases. Here, we review different mechanisms in EndMT and the role of EndMT in various cardiovascular diseases.

## 1. Introduction

Endothelial-to-mesenchymal transition (EndMT) plays a critical role in atrioventricular valve development; however, aberrant EndMT is reported in cardiovascular diseases (CVDs) such as atherosclerosis, pulmonary hypertension, valvular disease, fibroelastosis, and cardiac fibrosis. Accordingly, understanding the mechanisms behind the cause and/or effect of EndMT to eventually target EndMT appears to be a promising strategy for treating aberrant EndMT-associated diseases. Nevertheless, this approach is limited by a lack of information regarding precise functional and molecular pathways, cause and/or effect, and a lack of robust animal model and human data about EndMT in CVDs. Here, we review different mechanisms in EndMT and the role of EndMT in different CVDs. This review provides new insights into the molecular mechanisms and the importance of EndMT in cardiovascular pathophysiology.

## 2. Endothelial-to-Mesenchymal Transition (EndMT)

**Figure 1:**
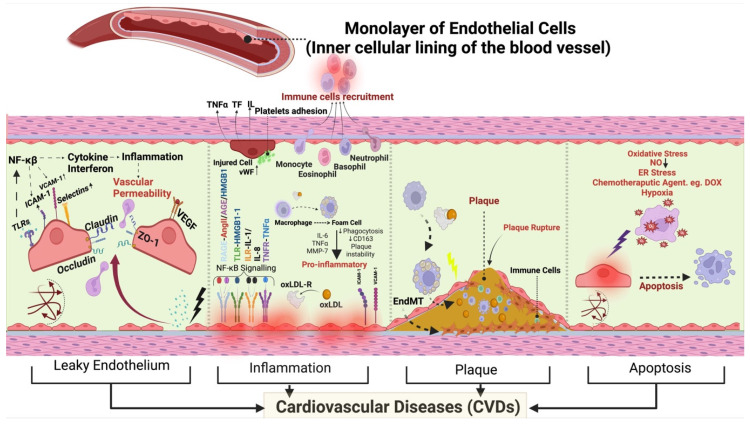
Endothelium and CVDs. Different mechanisms leading to leaky vasculature, increased inflammation, plaque development, or apoptosis in CVDs. The figure provides a detailed depiction of the interconnected mechanisms within endothelial dysfunction and their relevance to CVDs. It illustrates how the endothelium plays a crucial role in the pathogenesis of CVDs through various processes. The sequence begins with endothelial dysfunction leading to a leaky vasculature and increased vascular permeability, facilitated by TLR4 activation of the NF-κB pathway, upregulation of ICAM-1, VCAM-1, Selectins and subsequent cytokine release—all contributing to heightened inflammation. This cascade of events culminates in inflammation, affecting claudin-1 and ZO-1. The progression of inflammation exacerbates plaque development through the transformation of macrophages into foam cells, releasing pro-inflammatory cytokines, including IL-6, TNF-α, and MMP-7. Reduced phagocytosis and decreased CD163 expression highlight the impact of these processes on plaque instability within the vasculature. Additionally, upregulated vWF mediates endothelial activation and platelet adhesion, crucial for the inflammatory response. Moreover, vWF facilitates immune cell recruitment and acts as a signaling molecule, amplifying the inflammatory cascade. Plaque formation and rupture are highlighted as critical events triggered by endothelial dysfunction and oxLDL accumulation, contributing to the progression of CVDs. Finally, the figure illustrates apoptosis induced by various factors such as oxidative stress, hypoxia, reduced NO, ER stress, and chemotherapeutic agents and shear stress (as shown by red arrows pointing in the same direction, while others veer left or right, and some proceeding straight, capturing the dynamic nature of biomechanical forces acting on the endothelium). These factors contribute to apoptotic cell death within the vascular environment, emphasizing the diverse mechanisms through which endothelial dysfunction can lead to adverse outcomes in cardiovascular health, ultimately impacting the development and progression of various CVDs. TLR4: Toll-like Receptor 4, ICAM-1: Intercellular Adhesion Molecule-1, VCAM-1: Vascular Cell Adhesion Molecule-1, NF-κB: Nuclear Factor Kappa B, IL-6: Interleukin-6, TNF-α: Tumor Necrosis Factor- α, MMP-7: Matrix Metalloproteinase-7, CD163: Cluster of Differentiation 163, vWF: von Willebrand Factor, NO—Nitric Oxide, ER—Endoplasmic Reticulum, oxLDL: Oxidized Low-Density Lipoprotein, ZO-1: Zonula Occludens-1, EndMT: Endothelial-to-Mesenchymal Transition.

The endothelium is the inner luminal wall of blood and lymphatic vessels and is comprised of a monolayer of simple squamous endothelial cells. Endothelial cells are in direct contact with blood and its circulating biomolecules, thereby serving as sensors of, as well as responding to, hemodynamic, paracrine, and endocrinal changes. Ultimately, endothelial cells regulate the body’s systemic and vascular communication and homeostasis [1,2,3,4]. There are ~35 trillion endothelial cells in the human body occupying a surface area of >600 m^2^ to serve as a barrier between blood and the vascular matrix and to act as a mediator of gaseous/nutrient exchange between blood and tissues [4]. Endothelial cells facilitate angiogenesis and cell migration and maintain vascular tone [4,5]. However, oxidative, infectious pathogens, and/or metabolic insults injure endothelial cells [6], and injured endothelial cells are activated and leaky, contributing to inflammation, thrombosis, and vasoconstriction—overall impairing vascular tone [7]. Exposure to stressful stimuli for long periods results in endothelial dysfunction, which is characterized by inflammation, hypercoagulability, and dysregulated production of vasoactive factors or cell death, leading to different CVDs (Figure 1) [6].

Endothelial cells are plastic in physiological conditions, and stimuli such as blood flow can induce a phenotypic switch in endothelial cells where they lose their cobblestone-like morphology and gain a smooth mesenchymal-like and elongated fibroblast-like phenotype accompanied by the acquisition of motility and contractile properties. This phenomenon is called endothelial-to-mesenchymal transition (EndMT), where endothelial cells trans-differentiate into mesenchymal cells and gain invasive and migratory properties [8]. During EndMT, endothelial cells delaminate from their cell layer and invade the underlying tissue [8]. This trans-differentiation is accompanied by the loss of endothelial cell markers such as platelet-endothelial cell adhesion molecule (CD31), von Willebrand factor (vWF), vascular-endothelial cadherin (VE-Cadherin), and endothelial tyrosine kinase Tie-2 [8]. Conversely, there is a gain in mesenchymal markers, including α-smooth muscle actin (αSMA), N-Cadherin, vimentin (Vim), platelet-derived growth factor receptor alpha (PDGFRα), fibroblast-specific protein-1 (FSP-1), and collagens [8]. EndMT can be complete, partial [9], or transient [10].

In the embryonic heart, the presence of ciliated endothelial cells is restricted to areas of low and oscillatory flow, which is also marked by low expression of Krüppel-like Factor 2 (KLF2) [11,12,13]. The endocardial cushions are exposed to high shear stress and show elevated expression of KLF2 in the endothelial cells [12]. Interestingly, these endothelial cells are non-ciliated and undergo transforming growth factor beta (TGF-β)-driven EndMT, during which endothelial cells trans-differentiate to gain a mesenchymal phenotype and migrate into the cardiac jelly to form the primordia of cardiac valves [14]. This process occurs in the outflow tract and the atrioventricular canal in a spatiotemporally restricted manner, and it is thought to be initiated by inductive signals such as TGFβs, BMPs, and β-catenin from the myocardium [14,15,16,17,18].

**Figure 2:**
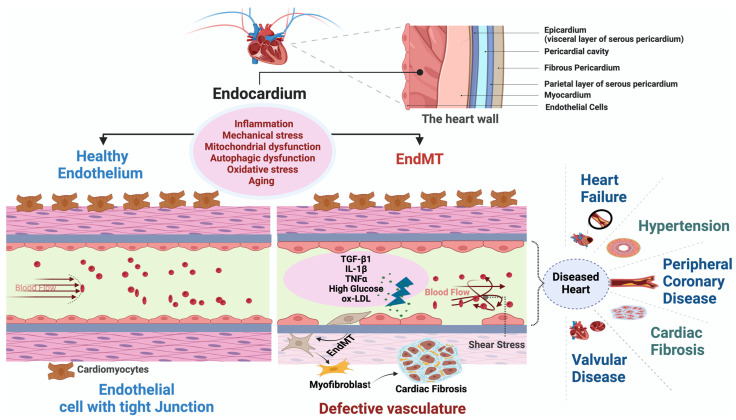
Figure depicting healthy endothelium and EndMT leading to different CVDs. Figure showing EndMT within endocardial endothelial cells, elucidating the contrast between the impermeable vascular structure of healthy endothelium and the disruptive impact of EndMT. Conversely, the EndMT, ignited by factors like shear stress, TGF-β, IL-1β, TNF-α, high glucose levels, and oxLDL, drives the transformation of active endothelial cells into myofibroblasts, promoting cardiac fibrosis. Beyond these factors, inflammation, aging, mitochondrial dysfunction, and autophagic dysfunction also contribute to the EndMT process. The appearance of a leaky endothelium signals the initiation of endothelial dysfunction within the endocardium, indicating a critical juncture in the path toward CVDs. This transformative journey not only disrupts endothelial integrity but heightens the risks of severe cardiovascular ailments such as heart failure, hypertension, peripheral coronary artery disease, cardiac fibrosis, and valvular abnormalities. These outcomes emphasize the intricate interplay between endothelial dysfunction, EndMT, and the spectrum of cardiovascular disorders, underscoring the necessity for a profound comprehension of these processes to drive targeted interventions and preserve cardiovascular well-being. EndMT: Endothelial-to-Mesenchymal Transition, TGF-β: Transforming Growth Factor-β, IL-1β: Interleukin-1β, TNF-α: Tumor Necrosis Factor-α, oxLDL: Oxidized Low-Density Lipoprotein.

In an adult heart, the endothelial lining of coronary blood vessels forms a protective barrier for cardiomyocytes and permits the delivery of paracrine factors to maintain cardiomyocyte health and function. The heart is one of the most aerobic organs, with >1 capillary next to every cardiomyocyte, and endothelial cells outnumber cardiomyocytes by ∼3:1 [19]. Cardiac endothelial cells directly influence myocardial contraction [19,20]. Thus, there exists an interdependency between endothelial cells and cardiomyocytes, which is crucial for the physiological transport of oxygen and nutrients and for paracrine communication between them [21,22]. Therefore, endothelial dysfunction within the heart results in extensive damage to the heart muscle by disrupting beneficial endothelial cell-cardiomyocyte cross-talk and by generating vessel leakiness that may cause direct damage to cardiomyocytes, ultimately leading to cardiac dysfunction [22]. Accordingly, loss of endothelial function in the heart in the form of EndMT is implicated in various CVDs, including heart failure, hypertension, coronary artery disease, cardiac fibrosis, and valvular disease (Figure 2) [20,22,23].

## 3. Mechanisms of EndMT

EndMT is a very complex process that is regulated by different mechanisms in various pathophysiological conditions in a context-dependent manner. Despite extensive research into the regulatory mechanisms of EndMT, details of the molecular alterations remain incompletely understood. In this section, we will discuss the different mechanisms implicated in the induction of EndMT.

### 3.1. TGF-β Signaling

**Figure 3:**
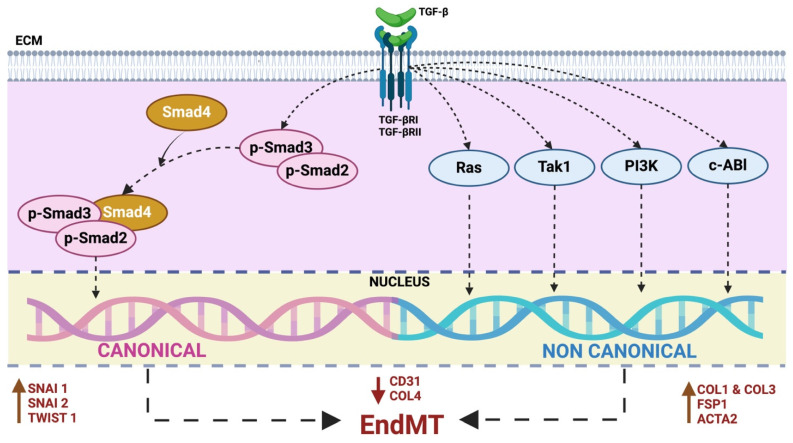
Canonical and noncanonical TGF- β signaling in EndMT. TGF-β homodimers interact with the TGF-β I/II receptor complex on the cell surface to initiate the canonical TGF-β pathway. This triggers the phosphorylation and activation of Smad2 and Smad3 proteins. Subsequently, the activated Smad2/3 complex with Smad4 translocate to the nucleus and activate transcription of genes involved in creating profibrotic extracellular matrix components such as Col4, Col1, Col3 and transcription factors like Snai1, Snai2, and Twist1. Noncanonical pathways involving kinases PI3K, Tak1, Ras, and c-Abl also influence EndMT. These pathways can lead to different cellular responses, independent of Smad2/3 activation, either by decreasing endothelial-specific gene transcription or increasing mesenchymal-specific gene expression. These pathways impact the expression of genes associated with EndMT, leading to variations in the transcription of markers like CD31, Col4, Col1, Col3, FSP1, and Acta2. Notably, these effects are also influenced by transcription factors such as Snai1, Snai2, and Twist1. TGF-β: Transforming Growth Factor-β, ECM: Extracellular Matrix, TGF-βRI/II: Transforming Growth Factor-β Receptor I/II, EndMT: Endothelial-to-Mesenchymal Transition, Col4: Collagen Type IV, Col1: Collagen Type I, Col3: Collagen Type III, PI3K: Phosphatidylinositol 3-Kinase, Tak1: TGF-β-activated kinase 1, c-Abl: Abelson Tyrosine-Protein Kinase 1, CD31: Cluster of Differentiation 31, FSP1: Fibroblast-Specific Protein 1, Acta2: Alpha Smooth Muscle Actin, Snail1: Zinc Finger Protein SNAI1, Snail2: Zinc Finger Protein SNAI2, Twist: Twist Family BHLH Transcription Factor 1.

Numerous studies have convincingly demonstrated TGF-β signaling-mediated induction of EndMT [21,23,24,25,26,27]. TGF-β is a multifunctional cytokine belonging to the transforming growth factor superfamily that includes mainly three different isoforms (TGF-β1 to 3), which serve as key initiators, yet they vary in potency in inducing EndMT [28,29]. Studies suggest that TGF-β1- and TGF-β3-induced EndMT may occur via paracrine stimulation by TGF-β2. The involvement of both canonical (Smad-dependent) and noncanonical (Smad-independent) TGF-β signaling pathways further complicates the picture. TGF-β homodimers activate canonical TGF-β signaling by binding to a heterodimeric TGF-β type I (TGF-βR1) and type II (TGF-βR2) receptor complex on the cell surface. Initially inactive, TGF-β calls for extracellular activation facilitated through integrins, in particular αvβ6 and αvβ1. Once activated, TGF-β binds to its heterodimeric receptor complex, leading to the phosphorylation of downstream effectors known as Smad proteins. Phosphorylated Smad2 and Smad3 form complexes with Smad4, which then translocate into the nucleus and regulate the transcription of target genes associated with EndMT, such as Snai1, Snai2, and Twist1 (Figure 3) [30,31]. Besides the classical Smad-dependent TGF-β pathway, TGF-β can also activate noncanonical pathways involving kinases such as Ras, Tak1, PI3K, and c-Abl. These pathways influence gene expression and cellular responses related to EndMT, providing alternative routes for TGF-β-mediated effects on endothelial cells (Figure 3) [32]. Understanding these pathways is essential for comprehensively deciphering TGF-β’s role in EndMT and its implications for cardiovascular health.

TGF-β signaling provides a link between cellular processes such as autophagy and EndMT. Autophagy is a cellular metabolic process wherein the cells break down unwanted biomolecules and organelles into basic components for their reuse [33,34]. Impaired autophagy has been attributed to CVDs [33,35], and it was demonstrated that genetic loss of endothelial autophagy via deletion of *autophagy-related gene 7* (*ATG7*) induced TGF-β-mediated EndMT in vitro and in vivo [34]. Endothelial cell-specific loss of ATG7 was associated with the upregulation of microRNA (miRNA) miR-378-3p, and inhibition of the gene expression of its target *Protein Disulfide Isomerase 4* (*PDIA-4*) in endothelial cells [36]. Endothelial-specific loss of PDIA-4 also inhibited autophagy and promoted TGF-β-mediated EndMT [37].

**Figure 4:**
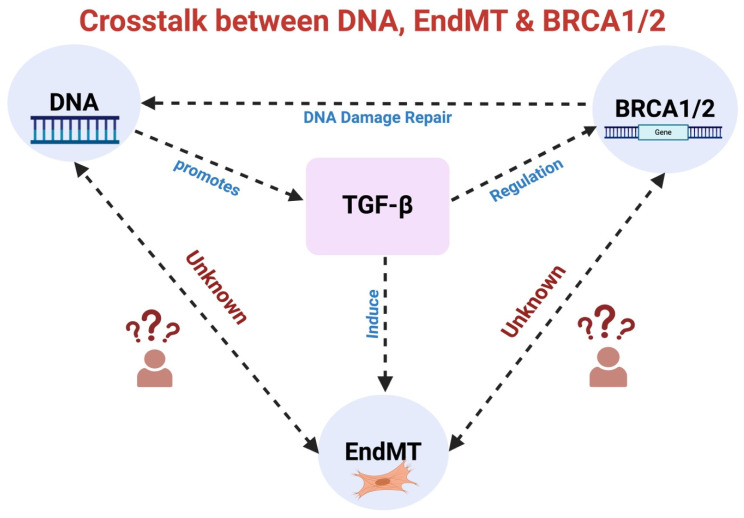
Unraveling the intricate interactions: TGF-β signaling, DNA damage repair, BRCA1/BRCA2, and EndMT. TGF-β signaling influences BRCA1/2 expression, pivotal in DNA damage repair, while DNA damage reciprocally enhances TGF-β signaling. Furthermore, TGF-β also induces EndMT. An established relationship exists between TGF-β and EndMT, TGF-β and DNA damage, and TGF-β and BRCA1/2. However, the direct effect of DNA damage and DNA damage repair molecules BRCA1/2 on EndMT remains unknown. TGF-β: Transforming Growth Factor-β, BRCA1/2: Breast Cancer Susceptibility Gene 1/2, EndMT: Endothelial-to-Mesenchymal Transition, DNA: Deoxyribonucleic Acid.

Additionally, breast cancer gene 1 and 2 (BRAC1/2) play a critical role in the DNA damage repair pathway; mutation in either BRCA1 or 2 is associated with an increased risk of breast and ovarian cancers [38] and CVDs [39]. DNA damage is also a common risk factor for CVD [40]. Interestingly, BRCA1, a key regulatory gene involved in the cell cycle and DNA repair, is known to interact with and regulate canonical TGF-β signaling [41,42]. Moreover, EndMT is reported in breast cancer [43], but whether there exists a link between BRCA genes and TGF-β-induced EndMT, particularly in the context of CVDs, remains unknown (Figure 4). Understanding how BRCA1 or BRCA2 mutation-associated genomic instability and tumor heterogeneity contribute towards TGF-β-induced EndMT could pave the way for the development of more effective treatment strategies aimed at targeting not only cancer cells and their tumor microenvironment but also different CVDS in BRCA1/2-mutant individuals.

### 3.2. Wingless/Integrated (Wnt) Signaling

The Wnt signaling pathway is also implicated in the induction of EndMT [44,45]. Wnt ligands are expressed and secreted widely by almost every cell type, including endothelial cells, with expression/secretion affected by different pathophysiological conditions [46]. Wnt signaling is initiated when Wnt ligands bind to membrane receptors like Frizzled (FZD) or other receptors like ROR1, ROR2, and RYK, triggering molecular events that regulate gene expression [47]. Based on the Wnt ligand–receptor interaction, canonical and noncanonical Wnt signaling pathways have been identified. The canonical Wnt signaling pathway, also known as the Wnt/β-catenin signaling pathway, prevents the degradation of β-catenin independent of its transcriptional regulation [48]. In this pathway, the Wnt ligand binds to FZD receptors and initiates intracellular events that include transcription of Snai1 and Snai2 genes crucial for the induction of EndMT [48]. Wnt/β-catenin signaling also provides a link between endothelial primary cilia and EndMT. Primary cilia are cellular protrusions that serve as mechanosensors for fluid flow. In endothelial cells, they function by transducing local blood flow information into functional responses, such as expression of shear-sensitive transcription factors [49] and nitric oxide production [50]. Studies demonstrate that endothelial cilia regulate Wnt/β-catenin signaling [51,52,53]. Wnt/β-catenin signaling interacts with TGF-β signaling and loss of endothelial cilia is associated with an increased translocation of β-catenin to the nucleus, which induces EndMT [54]. Notably, loss of intraflagellar transport protein 88 (Ift88), a protein essential for cilia formation, led to TGF-β-mediated EndMT; however, β-catenin was not affected [50].

On the other hand, noncanonical Wnt signaling pathways are independent of β-catenin and encompass diverse cascades, with the most studied being the calcium (Wnt/Ca^2+^) and planar cell polarity (Wnt/PCP) pathways [55]. Unlike the well-known β-catenin-driven Wnt pathway, the Wnt/Ca^2+^ pathway starts with the release of Ca^2+^ inside the cell, which then activates protein kinase C (PKC) and Ca^2+^/calmodulin-dependent protein kinase II (CAMKII) [56,57]. There are no reports linking the Wnt/Ca^2+^ pathway with EndMT. On the other hand, endothelial cell polarity holds significant importance in the cardiovascular system in maintaining the structural integrity of blood vessels [58]. Mechanical factors such as shear stress play a significant role in regulating cell polarity pathways within endothelial cells [59]. Changes in shear stress, caused by variations in blood flow patterns, can activate signaling pathways involved in cell polarity, such as the Wnt/PCP pathway, and can induce EndMT [55,60]. The precise molecular mechanisms underlying how disturbed shear stress disrupts Wnt/PCP signaling and promotes EndMT remain unclear and warrant further investigations.

### 3.3. Notch Signaling

Notch signaling’s role in EndMT was initially observed in vitro in cultured endothelial cells [61] and later confirmed in cardiac valve development in vivo [62]. The Notch signaling pathway is a highly conserved cell signaling system that mediates a distinctive developmental role by linking the fate of one cell with that of an adjacent cell through direct physical interactions between the Notch receptors; Notch 1–4 on one cell and membrane-bound ligands (Delta-like 4, Jagged 1) expressed on a neighboring cell, leading to a sequential proteolytic cleavage of the Notch intracellular domain (NICD) [63,64]. The NICD then translocates into the nucleus, where it interacts with transcription factors such as CSL/RBP-Jκ, activating the expression of genes involved in EndMT, including Snai1 and Snai2 [65,66,67]. Additionally, in diseases like atherosclerosis and cardiac fibrosis, dysregulated Notch signaling can lead to EndMT through altered expression of Notch ligands and receptors, leading to downstream transcriptional changes associated with mesenchymal transition [68]. Moreover, in pulmonary fibrosis, C-X-C chemokine receptor type 7 (CXCR7) has been found to mitigate EndMT by suppressing the Jag1-Notch pathway, which is implicated in promoting EndMT [69]. However, contrary studies witnessed endothelial cell-specific inhibition of the Notch pathway in accelerated EndMT and worsening fibrosis [70]. Nonetheless, Notch signaling’s involvement in EndMT and consequent fibrosis underscores its intricate role in tissue homeostasis and pathology with potential therapeutic implications.

## 4. Epigenetic Regulation of EndMT

Epigenetics has gained recognition for its role in the regulation of gene expression without altering the underlying DNA sequence and operating via DNA methylation, histone modifications, and noncoding RNA regulation [33]. Initially linked to cellular differentiation, it is now known to play critical roles in almost every pathophysiological condition, including EndMT [71]. In general, DNA/histone modifications directly alter chromatin structure, impacting transcription, while noncoding RNAs modulate transcriptional, post-transcriptional, and translational processes. There exists reciprocal regulation among these epigenetic pathways, with DNA/histone modifications influencing noncoding RNA activity and vice versa. This interplay dictates the balance of gene expression, highlighting the complexity of epigenetic regulation in shaping cellular phenotypes and responses [33].

### 4.1. Histone Modification

Histone proteins, which are integral for DNA packaging, undergo histone modifications, encompassing diverse post-translational modifications such as methylation, acetylation, and phosphorylation that activate or repress transcription by altering chromatin structure [72]. In EndMT, histone post-translational modifications (PTMs), notably methylation and acetylation of lysine residues, intricately regulate chromatin architecture and gene expression. Trimethylation of histone H3 at lysine 4 (H3K4me3) and lysine 27 (H3K27me3) typically activate and suppress transcription, respectively. Conversely, histone lysine acetylation generally promotes transcription by enhancing chromatin accessibility. This balance is governed by histone-modifying enzymes, including methyltransferases and acetyltransferases (writers), and demethylases and deacetylases (erasers). Notably, enhancer of zeste homolog 2 (EZH2), a histone methyltransferase, catalyzes H3K27 trimethylation associated with transcriptional repression, while histone deacetylases (HDACs) remove acetyl groups, leading to chromatin condensation and gene repression. Dysregulation of these enzymes disrupts normal gene expression, contributing to EndMT and associated diseases such as cancer and cardiovascular disorders. Targeting EZH2 and HDACs presents potential therapeutic avenues for diseases driven by aberrant epigenetic regulation [73,74]. EZH2 plays a crucial role in EndMT regulation, especially during heart development, where HDAC3 recruits EZH2 to turn off the activity of TGF-β1 and thereby inhibits EndMT [75]. Another recent study by Glaser et al. shows that increased histone demethylase, such as lysine demethylase 4B (KDM4B), expression/activity promoted EndMT. Inhibition of KDM4B activity prevented the demethylation of repressive H3K9me3 within the promoters of genes controlling the mesenchymal phenotype and EndMT, consequently suppressing EndMT [76]. Histone deacetylation, mediated by HDACs, is known to play a role in EndMT [77]. Lecce et al. investigated HDAC9’s role in EndMT [78]. Ex vivo, HDAC9 knockout in endothelial cells prevented EndMT and maintained an endothelial-like phenotype. In vivo, atherosclerosis-prone mice with endothelial-specific HDAC9 knockout showed reduced EndMT, smaller plaque areas, and improved plaque composition [78]. Additionally, a study by Murugavel et al. showed that valproic acid (VPA), an HDAC inhibitor treatment for endothelial cells, leads to TGFβ-mediated EndMT [71].

### 4.2. DNA Methylation

DNA methylation is a dynamic epigenetic modification involving the addition of a methyl group to cytosine residues within CpG dinucleotides, primarily situated in the promoter regions [79]. Methylated CpG sites are recognized by chromatin-modifying enzymes, which hinder the binding of transcription factors to gene promoters, thus repressing transcriptional activity. Maintenance of DNA methylation patterns during cellular replication is orchestrated by DNA methyltransferases (DNMTs), ensuring faithful transmission of epigenetic information to daughter cells and playing fundamental roles in cellular differentiation, development, and pathogenesis [80]. Studies have demonstrated abnormal methylation induced by TGF-β1 in mouse models of cardiac fibrosis and clinical observations in heart failure patients with reduced Ras protein activator-like 1 (RASAL1) expression, compromising its role in suppressing EndMT. This disruption initiates a cascade leading to EndMT progression [81]. In another cardiac fibrotic condition, hypermethylation of the BMP7 gene was observed, which is a crucial EndMT inhibitor [82]. These studies clearly demonstrate the role of histone and DNA modification in EndMT.

### 4.3. Noncoding RNAs

Noncoding RNAs comprise small noncoding RNAs (sncRNAs; <200 nucleotides long) and long noncoding RNAs (lncRNAs; >200 nucleotides long) that play pivotal roles in epigenetic regulation [83,84]. Noncoding RNAs regulate protein-coding genes by acting as intermediaries in gene expression despite not encoding proteins themselves. They influence gene activity through mechanisms such as binding to messenger RNA (mRNA), guiding mRNA degradation, or inhibiting translation [84,85]. Moreover, noncoding RNAs also interact with DNA or chromatin, modifying chromatin structure and recruiting regulatory proteins. This intricate regulation fine-tunes gene expression for the regulation of complex cellular processes.

Noncoding RNAs, particularly microRNA (~22 nucleotides long) and lncRNAs, exert fine-tuned control over EndMT by targeting key regulators of endothelial markers and mesenchymal transition [86,87]. Studies suggest that microRNAs miR-21 and miR-29 have opposing effects in EndMT-related conditions. Specifically, increased miR-21, abundant in cardiac fibroblasts, promoted EndMT [88]. In contrast, reduced miR-29 levels near the infarct zone post-myocardial infarction lead to increased expression of extracellular matrix components such as collagen types, fibrillin, and elastin, indicating increased EndMT [89]. Research conducted on lncRNAs is not as widespread compared to miRNA, with only a few lncRNAs known to regulate EndMT, one of them being metastasis-associated lung adenocarcinoma transcript 1 (MALAT1), which is identified as a driver of EndMT. It does so by binding competitively to miR-145, known for its ability to halt TGF-β1-induced EndMT by directly targeting TGF-βRII and Smad3. Acting as a sponge for miR-145, MALAT1 disrupts its suppressive action, thereby promoting EndMT [90]. These revelations underscore the intricate regulatory web spun by noncoding RNAs, urging further exploration to unveil their mechanisms and therapeutic potentials in combating EndMT-associated pathological conditions [91].

## 5. EndMT in Cardiovascular Diseases

**Figure 5:**
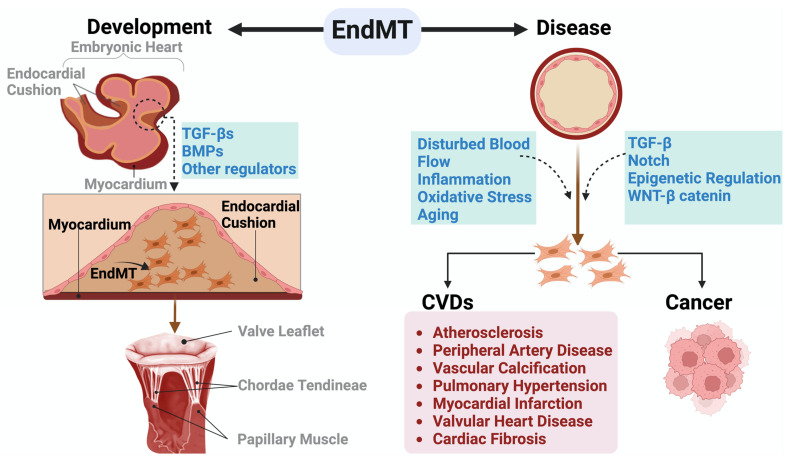
EndMT in development and disease. During embryonic development, specific endothelial cells within the AV canal engage in EndMT, facilitating the creation of endocardial cushions essential for the proper development of the AV valve. As the embryo matures, these endocardial cushions undergo remodeling to form durable valve leaflets and the supporting chordae tendineae. In adult life, EndMT plays a crucial role in the initiation and progression of various CVDs and cancer. EndMT: Endothelial-to-Mesenchymal Transition, CVDs: Cardiovascular Diseases.

Aberrant EndMT is reported in the progression and development of cancer as well as CVDs such as atherosclerosis, peripheral artery disease, vascular calcification [92], pulmonary hypertension, valvular disease, myocardial infarction, and cardiac fibrosis (Figure 5) [93,94,95,96]. The role of EndMT in CVDs is discussed in detail in this review, and many detailed reviews are available for the role of EndMT in cancer [97].

### 5.1. EndMT in Atherosclerosis

Atherosclerosis is a widespread chronic inflammatory disease in which plaque (fatty deposits) build up inside arteries, causing the narrowing of arteries leading to reduced blood supply to tissues of vital organs. Atherosclerosis can affect arteries supplying blood to heart ventricles and extremities, causing coronary artery disease (CAD) and peripheral artery disease (PAD), respectively. Studies implicate EndMT in the development and progression of atherosclerosis [98]. Various factors such as inflammation, shear stress, and oxidative stress in the form of oxidized-low-density lipoprotein (oxLDL) are known to play a role in atherosclerosis and also promote EndMT [98,99,100,101,102]. Inflammation is a key feature of atherosclerosis that involves cytokines like IL-1β, TNF-α, and IFN-γ, which encourage cells to adopt mesenchymal characteristics promoting EndMT via TGF-β-dependent and -independent pathways [99]. While temporary inflammation typically does not trigger EndMT, persistent inflammation due to factors like high cholesterol or chronic irritation can activate this process by disrupting a protective cellular signaling pathway known as FGF signaling. Once EndMT is initiated, it exacerbates inflammation, creating a harmful cycle where each amplifies the other [103]. Interventions targeting specific inflammatory cytokines may not effectively halt this cycle; for instance, blocking IL-1β with canakinumab yielded mixed results and posed serious risks, including increased susceptibility to fatal infections. To reverse atherosclerosis, it is imperative to address the underlying inflammation and EndMT cycle, emphasizing the need to identify and tackle the root causes of treatment resistance [104].

Additionally, the region with reduced fluid shear stress characterized by intricate directional fluctuation throughout the cardiac cycle, known as “disturbed shear stress (DSS)”, significantly influences vascular development and remodeling, EndMT, and atherosclerosis progression. Endothelial responses to shear stress determine vascular function in both health and disease. DSS occurs in arterial regions with branching or sharp curvature, leading to irregular flow patterns and moderate activation of inflammatory pathways in endothelial cells that sensitize them to other inflammatory mediators. Conversely, physiological shear stress suppresses inflammation and cytokine responses [105]. The role of DSS-associated EndMT in atherosclerosis is further supported by research conducted by Chen et al. (2015) [94]. In their study, both inflammatory cytokines and oscillatory shear stress were found to diminish endothelial FGFR1 expression while activating TGF-β signaling in cultured human endothelial cells [94]. This interplay prompted an investigation into the relationship between disrupted FGF endothelial signaling and the progression of EndMT in atherosclerosis. In their study involving atherosclerotic (ApoE^−/−^) mice, Chen et al. (2015) deliberately deleted endothelial FGF receptor substrate 2 α (FRS2α) [94]. Remarkably, these double-knockout mice exhibited extensive EndMT and accelerated atherosclerosis development compared to ApoE^−/−^ mice when subjected to a high-fat diet [94]. Further substantiating these experimental findings, Chen et al. (2015) conducted an analysis of the left main coronary arteries of 43 patients with coronary disease [94]. Their study unveiled a robust correlation, linking the extent of coronary atherosclerosis with diminished endothelial FGFR1 expression, activated endothelial TGF-β signaling, and a heightened prevalence of EndMT [94]. These comprehensive findings underscore the critical connection between diminished protective endothelial FGFR signaling, the initiation of EndMT, and the relentless progression of atherosclerosis [94]. Additionally, it is worth noting that plaque erosion, a significant occurrence in atherosclerosis, arises when arterial plaque ruptures without damaging the fibrous cap. This exposes the inner plaque surface directly to blood, fostering thrombosis and precipitating heart attacks. Intriguingly, plaque erosion predominantly arises in regions of DSS, such as where arteries bifurcate or bend [106]. This correlation begs the question: Does EndMT, a process implicated in atherosclerosis, contribute to endothelial vulnerability in these areas, potentially predisposing to plaque erosion? Further research elucidating the interplay between EndMT and plaque erosion could unveil critical insights into preventing atherosclerotic events. Furthermore, in both human endothelial cells and mouse aortae, unidirectional laminar flow upregulates tenascin-X (TN-X) expression via KLF4, a process not observed with DSS. Mice lacking endothelial TN-X (EC-Tnxb-KO) exhibit heightened endothelial TGF-β signaling, along with increased expression of EndMT and inflammatory markers [107]. EC-Tnxb-KO mice, especially when bred with LDL receptor (LDLR)-deficient mice and fed a high-fat diet, display advanced atherosclerotic lesions. However, treatment with an anti-TGF-β antibody or additional loss of endothelial TGF-βRI and II in EC-Tnxb-KO mice normalizes TGF-β signaling and prevents EndMT. In vitro experiments reveal that TN-X, via its fibrinogen-like domain, directly binds to TGF-β, interfering with its interaction with the TGF-β receptor. These findings elucidate TN-X as a pivotal regulator of flow-mediated suppression of EndMT, endothelial inflammation, and atherogenesis, operating through its interaction with and inhibition of TGF-β. This discovery unveils a novel mechanism governing flow-dependent modulation of vascular TGF-β, offering potential avenues for devising novel approaches to combat vascular inflammation and atherosclerosis [107].

A recent study reveals a metabolic shift in endothelial cells, driven by increased acetate production from glucose, as the foundation of TGF-β-induced EndMT [108]. These findings highlight the role of metabolic regulation in EndMT initiation and progression [108]. EndMT’s role in driving atherosclerosis forward makes it a potential target for anti-atherosclerosis treatments. Accordingly, hydrogen sulfide (H_2_S) has been found to have protective effects on the cardiovascular system due to its ability to reduce inflammation, oxidative stress, foam cell formation, regulate ion channels, and improve cell adhesion and endothelial function. However, the exact mechanism by which H_2_S works against atherosclerosis and its impact on EndMT are still unclear [109]. As the role of EndMT in atherosclerosis is unveiled, the exploration transcends into the realm of vascular calcification, a crucial aspect for understanding its correlation with the progression of atherosclerosis, shedding light on the complex pathways influencing cardiovascular health.

### 5.2. EndMT in Vascular Calcification

The hallmark of atherosclerosis is the formation of plaques within arterial walls, comprised of cholesterol, immune cells, and cellular debris. As the disease progresses, these plaques undergo calcification, a process where calcium deposits accumulate within the arterial wall. Vascular calcification (VC) denotes the accumulation of calcium–phosphate complexes within the vasculature, a phenomenon implicated in aging, as well as various pathological conditions such as hypertension, chronic kidney disease, diabetes, and certain hereditary disorders [110,111,112] and poses a significant threat to cardiovascular health. Traditionally, research primarily focused on the role of vascular smooth muscle cells (VSMCs) in this process. However, a surge in VC studies reveals endothelial cells as another significant contributor to the development and progression of VC through EndMT [113,114,115]. During VC, endothelial cells transform into bone progenitor cells via EndMT [115], which then migrate to the vascular wall, contributing to VC. These aberrant cells contribute to vessel stiffness by synthesizing proteins that promote calcification, elucidating the intricate nature of VC pathogenesis. Additionally, endothelial cells secrete bone growth factors such as bone morphogenetic protein (BMPs), inflammatory mediators, and cytokines, indirectly promoting osteoblast differentiation, particularly in conditions like hyperlipidemia and diabetes [116]. Enhanced BMP signaling is particularly evident in Matrix Gla protein (Mgp)^−/−^ knockout mice, a recognized model of VC [115]. This transformation is triggered by enhanced BMP signaling, inducing EndMT and rendering endothelial cells adaptable to osteoblastic traits, thus contributing to VC. Moreover, heightened BMP activity prompts specific serine proteases in endothelial cells, facilitating EndMT and migration of endothelial cell-derived osteoblast-like cells to the aorta wall. Pedigree tracing in calcified blood arteries reveals labeled endothelial cells expressing osteoblast markers, underscoring endothelial cells’ osteoblastic transformation via EndMT [117].

In a study investigating endothelial contribution to VC, aortic endothelial cells in wild-type and Mgp^−/−^ mice were examined. Mgp^−/−^ mice exhibit notable vascular abnormalities, including aortic calcification, tachycardia, elevated pulse wave velocity, and endothelial dysfunction [115]. Microscopic examination revealed substantial endothelial alterations, with a mixture of abnormal cells, including chondroblast-like cells, replacing normal endothelial cells. Molecular analysis demonstrated increased expression of endothelial lineage markers, and immunostaining revealed co-expression of endothelial markers with osteogenic markers in calcified areas of the aorta. Additionally, flow cytometry analysis showed a significant proportion of cells co-expressing endothelial and bone markers in Mgp^−/−^ aortas. These findings suggest an active involvement of the EndMT in VC, with endothelial cells potentially acquiring osteogenic characteristics [115]. TGF-β signaling further fuels this transition by activating downstream Smad pathways that culminate in the phenotypic switch necessary for VC [118]. Additionally, Wnt/β-catenin signaling facilitates EndMT through the nuclear translocation of β-catenin, thereby regulating genes pivotal in endothelial cell transformation and calcification [119]. Notch signaling also orchestrates EndMT involved in VC [116]. Furthermore, inflammatory signaling pathways, including cytokines IL-1β and TNF-α, induce EndMT by activating intracellular cascades such as NF-κB, underscoring the role of cytokines in promoting the transition to a mesenchymal phenotype associated with VC [120,121,122]. Collectively, these signaling pathways intricately regulate EndMT, unveiling promising avenues for therapeutic interventions targeting VC in CVDs.

### 5.3. EndMT in Peripheral Artery Disease

Peripheral artery disease (PAD) is characterized by stenosis in arteries supplying blood to the lower extremities due to plaque build-up [123]. One of the critical manifestations of atherosclerotic PAD is critical limb ischemia (CLI), which produces intractable lower limb pain, non-healing ulcers, and tissue necrosis requiring limb amputation [124,125]. There is potential evidence suggesting a relationship between EndMT and PAD. A study by Chevalier et al. assessed two groups of individuals’ intramuscular arteriolar in lower limb specimens with and without CLI and observed 1.8-fold higher arteriolar densities among patients with CLI because of bulky, re-oriented endothelial cells expressing upregulated N-cadherin, S100A4, and EndMT-related transcription factor Snai1—overall promoting EndMT that results in stenosis of 33% small arterioles and occlusion of 9% arterioles [95]. The presence of TGF-β1 in smooth muscle cells (SMCs) in the muscles of patients with CLI is reported [126,127]. This finding aligns with existing research connecting TGF-β in SMCs with vascular diseases, including PAD [128,129]. These observations and identification of phosphorylated Smad2/3 in the nucleus of endothelial cells of narrowed arterioles suggest TGF-β1-driven SMC-endothelial cell axis leading to EndMT in arterioles and resulting in constricted blood vessel lumens affecting arteriolar integrity. Apart from this, there is potential for abnormal hemodynamics in the microvasculature to influence change in the endothelial phenotype and cause EndMT [130]. Currently, PAD is primarily treated using medication and surgery, but some patients who are unable to undergo revascularization due to complications may face limb amputation [123]. Hence, redirecting focus towards alternative therapeutic avenues becomes imperative. In this context, EndMT emerges as a compelling target warranting further exploration.

### 5.4. EndMT in Pulmonary Hypertension

Pulmonary hypertension (PH) is a complex and progressive disorder characterized by elevated mean pulmonary arterial pressure exceeding 20 mmHg within the pulmonary artery. This condition encompasses a diverse spectrum of cardiopulmonary pathologies, classified into five distinct groups: (1) pulmonary arterial hypertension (PAH), (2) PH associated with left heart disease, (3) PH associated with lung disease and/or hypoxia, (4) chronic thromboembolic PH, and (5) PH with unclear multifactorial mechanisms [131]. Despite advances in our understanding of PH pathophysiology, current treatment options remain limited, emphasizing the need for novel therapeutic strategies. PH can lead to heart failure and eventually death if left untreated.

EndMT is triggered by various PH-associated factors present in the pulmonary vascular microenvironment, including inflammation, oxidative stress, and mechanical stress [132]. Accordingly, EndMT has emerged as a crucial biological process implicated in the pathogenesis of PH [132], and accumulating evidence suggests that EndMT-derived mesenchymal-like cells contribute to vascular remodeling [132], fibrosis [133], and endothelial dysfunction [132], leading to pulmonary vascular dysfunction and elevated pulmonary artery pressure [132]. The Qiao et al. research team was the first to observe EndMT by confirming the presence of endothelial cell-specific markers PECAM-1 and vWF alongside α-SMA, a marker of SMC, in the cells within blood vessel walls of PAH patients [134]. In a subsequent study, Ranchoux and colleagues confirmed these findings by examining lung tissue from PAH patients, showing endothelial cells co-expressing CD31 along with α-SMA [135]. Moreover, using a rat model of PH, Ranchoux et al. showed thickening of the walls of pulmonary arteries, which was accompanied by increased levels of transcription factor Twist1 and a mesenchymal marker called phospho-vimentin, indicating a potential link between impaired BMPR2 signaling and the occurrence of EndMT in PH [135]. Twist1, a transcription factor implicated in EndMT, was found to be upregulated and activated under conditions of low oxygen, or hypoxia, led to enhanced TGF-β signaling, including the expression of TGF-β receptor TGF-βRII, ultimately resulting in the induction of EndMT in human pulmonary arterial endothelial cells (PAECs) and notably introducing a mutated form of Twist1, prevented these hypoxia-induced changes associated with EndMT [28]. Along with dysregulation of TGF-β and BMP signaling pathways, inflammatory cytokines, such as IL-6 and TNF-α, are also shown to promote EndMT in response to vascular injury and inflammation in PH [136].

Furthermore, DSS and high shear stress (HSS) both induce EndMT and are associated with certain heart conditions like PAH. To understand this better, endothelial cells from individuals with heart conditions such as PAH were studied, which showed BMPR2-mediated EndMT following HSS [137]. Another protein called ETS-related gene (ERG) was reduced in these endothelial cells under HSS, which seems to play a role in triggering EndMT [137]. Restoring ERG levels reduced the harmful effects of HSS, suggesting a potential treatment approach for heart conditions related to blood flow abnormalities [137]. Given the significant role of EndMT in PH, targeting EndMT has emerged as a promising therapeutic approach. Strategies aimed at inhibiting key signaling pathways involved in EndMT, such as TGF-β and BMP inhibitors, hold potential for preventing or reversing vascular remodeling and fibrosis in PH. Furthermore, modulation of endothelial cell phenotype through the use of endothelial-specific transcription factors represents an innovative therapeutic avenue to treat or prevent PH [136].

### 5.5. EndMT in Myocardial Infarction

Myocardial infarction (MI), commonly known as a heart attack, is characterized by the irreversible death of heart tissue due to plaque rupture in the coronary artery, causing prolonged oxygen deprivation, leading to acute inflammation, cardiomyocyte death, and scar formation [138]. Swift recognition and intervention are crucial in mitigating the severe consequences of MI, and if left untreated, it leads to heart failure and death. Following MI, EndMT generates mesenchymal cells that aid in heart tissue repair but can also lead to harmful fibrosis, excessive scar tissue, and compromised heart function, complicating post-MI tissue healing [139]. Post-MI EndMT is marked by the emergence of CD31+/αSMA+ double-positive cells, peaking at day 7 post-MI. This transition involves increased expression of mesenchymal markers and canonical Wnt signaling activity, suggesting a role in granulation tissue formation [139]. EndMT is triggered by factors present during MI that include inflammation, oxidative stress, mechanical strain, and activating pathways such as TGF-β and Notch that contribute to endothelial cell transformation into mesenchymal-like cells, and these transformed cells can then migrate into the myocardium and contribute to fibrosis by producing extracellular matrix proteins like collagen [140].

Previous research has shown that TGF-β signaling is a central mediator of EndMT induction in myocardial fibrosis post-MI. Inhibition of TGF-β signaling using recombinant human BMP-7 attenuated EndMT and reduced myocardial fibrosis in a mouse MI model, emphasizing the therapeutic potential of targeting this pathway [133]. The study by Xu et al. revealed the transition of human coronary artery endothelial cells into mesenchymal cells under hypoxic conditions through the activation of the Snail transcription factor by HIF-1 [141]. This EndMT process was associated with specific genetic pathways and observed in murine cells after MI or exposure to hypoxia [141]. Bulk RNA sequencing performed on murine endothelial cells following myocardial infarction or exposure to tissue hypoxia displayed the expression of mesenchymal genes [142]. These findings show that hypoxia plays a crucial role in endothelial cell plasticity and highlight the significance of EndMT in cardiovascular pathologies.

Current therapeutic methods for MI, including drug intervention, reperfusion therapy, medical instrument implantation, and organ transplantation, are hindered by limitations such as invasiveness, donor organ scarcity, thrombosis, vascular restenosis, and immune rejection [143,144,145]. Therefore, there is an urgent need to develop new therapeutics with low cost and high efficacy. Targeting EndMT presents a promising approach to reduce fibrosis and enhance heart recovery, offering a novel therapeutic strategy for managing this prevalent cardiac ailment.

### 5.6. EndMT in Valvular Heart Disease

Valvular heart disease, affecting the heart’s crucial valves, disrupts efficient blood flow regulation. The heart’s four valves—tricuspid, pulmonary, mitral, and aortic—are vital for ensuring that blood moves correctly through its chambers, maintaining optimal circulation [146]. If left untreated, valvular heart disease can lead to severe complications, including heart failure, arrhythmias, and sudden cardiac death. Various factors contribute to valvular heart disease, including rheumatic fever [147], endocarditis [148], heart attack [149], abnormality of the heart muscle [150], and atrial fibrillation [151]. Under normal physiological circumstances, endothelial cells undergo EndMT for proper cardiac valve development in the embryonic heart [152]. Interestingly, studies have revealed that endothelial cells lining the adult heart valve surface can also undergo EndMT [153,154]. Accordingly, the potential role of EndMT in mitral valve changes associated with mitral valve prolapse was explored. Remarkably, patients undergoing mitral valve replacement for mitral valve prolapse exhibited elevated levels of circulating osteoprotegerin. Subsequent experiments demonstrated that osteoprotegerin induces EndMT in cultured endothelial cells isolated from pathological mitral valves. Intriguingly, mitral valve endothelial cells undergoing EndMT were found to produce and secrete osteoprotegerin, suggesting an autocrine pathogenetic mechanism for EndMT in mitral valve prolapse [155]. Other studies have further illuminated the intricate role of EndMT in the progression of valvular diseases. Specifically, research has revealed the differentiation of mitral valve endothelial cells into mesenchymal cells, some of which exhibit osteogenic potential, thus implicating them in the induction of vascular calcification [156]. This process of vascular calcification, in turn, can contribute to the narrowing of the valve opening, ultimately culminating in aortic stenosis, which is frequently observed in clinical settings [157]. In another recent study, mitral valve leaflets from sheep with inferior MI displayed signs of EndMT. Post-MI plasma induced EndMT marker expression and enhanced migration of mitral valve endothelial cells (VECs), while sham plasma did not show any effect. This effect coincided with a significant decrease in the Wnt signaling antagonist secreted Frizzled related protein (sFRP3) in post-MI plasma. Additionally, mitral VECs exposed to post-MI plasma showed upregulated forkhead box M1 (FOXM1) expression, and blocking FOXM1 reduced EndMT transcripts in mitral VECs treated with post-MI plasma. These results highlight potential therapeutics for addressing EndMT-related pathologies post-MI [45]. Collectively, these studies underscore the pivotal role of endothelial cell plasticity in the pathogenesis of valvular diseases, providing novel insights into potential therapeutic targets for mitigating disease progression.

### 5.7. EndMT in Cardiac Fibrosis

The accumulation of activated myofibroblasts in affected tissues remains a crucial determinant of the prognosis, the efficacy of clinical approaches, and the mortality rate of fibrotic disorders [158,159]. Previous immunofluorescence confocal microscopy and endothelial cell-lineage-tracing studies in disease models in vitro and in vivo, have suggested that EndMT facilitates the generation of activated myofibroblasts in various organs [24,159,160]. Accordingly, Hashimoto et al., using bleomycin-induced lung fibrosis mice, demonstrated that ~16% of lung fibroblasts in these mice were derived from endothelial cells, suggesting that EndMT may be a source of interstitial fibroblasts in pulmonary fibrosis [161]. Zeisberg et al., using chronic kidney disease murine models in an extensive study examining the role of EndMT in fibrosis, also showed that 30–50% of myofibroblasts in the animal’s fibrotic kidneys were of endothelial origins [133].

Diastolic dysfunction is a significant cause of heart failure and is associated with cardiac fibrosis, which underlies the increased stiffness of the heart that is common in patients with advanced cardiomyopathy [162,163,164]. Cardiac fibrosis is characterized by excessive extracellular matrix deposition mediated by an increased fibroblast population, resulting in disrupted microvascular and myocardial structures [159,165]. Specific anti-fibrotic therapies are not currently available in clinics [166] and the pathological origin of these fibroblasts in the adult heart remains under investigation [133]. Adult fibroblasts are known to be derived directly from the embryonic mesenchymal cells, a process induced by the proliferation of resident fibroblasts [133,165]. Recently, tissue fibroblast accumulation has been attributed to endothelial cells via their capacity to undergo EndMT, during which resident endothelial cells delaminate from an organized cell layer (loss-of cell–cell junction) and invade the underlying tissue (gain-of migratory ability) as they acquire a mesenchymal phenotype detected by the down- and up-regulation of endothelial and mesenchymal markers, respectively [167,168]. Accordingly, these EndMT-derived cell populations share fibroblast characteristics, contributing to matrix deposition, tissue remodeling, and fibrosis [133,169,170,171]. Zeisberg et al. confirmed this observation in a lineage tracing study and estimated that about one-third (27–35%) of the total fibroblasts in the fibrotic lesion were of endothelial origin, suggesting that endothelial cells contribute to the cardiac fibroblast pool via EndMT [133].

TGF-β signaling is implicated in the pathogenesis of cardiac fibrosis by stimulating cardiac fibroblasts to produce collagens [172]. A study demonstrated that a Smad3-dependent TGF-β signaling pathway drives cardiac fibrosis via inducing EndMT [172]. Interestingly, Rosa26/Tie2 reporter mice with systemic administration of BMP7, an antagonist of EndMT known to counteract TGF-β’s action, exhibited a significant reduction in the number of fibroblasts co-expressing endothelial-derived LacZ, along with reduced extracellular matrix deposition and improved heart function [133]. However, another study by Moore-Morris et al. later confirmed the role of resident fibroblast rather than EndMT in cardiac fibrosis [173]. This study was supported by a study by Singh et al., where the loss of endothelial cilia-associated TGF-β-mediated EndMT did not exacerbate cardiac fibrosis in mice [50]. Although controversial, EndMT has been implicated in different stages of cardiac fibrosis, in addition to the contribution of the proliferation of resident cardiac fibroblasts [167,173].

## 6. Conclusions

Regardless of the critical role of EndMT in embryonic development, EndMT is mainly detrimental in adult organs or vasculature that contributes towards the development and progression of CVDs. This review provides recent advances in understanding the regulation and different mechanisms of EndMT in CVDs. Given the critical role of EndMT in CVDs, modulating EndMT may provide an exciting and novel therapeutic avenue to prevent and/or treat these diseases. Accordingly, currently, there are strategies being considered to modulate EndMT in pathological conditions; however, a number of obstacles remain to be overcome prior to realizing the full therapeutic potential of modulating EndMT. These include the mechanisms involved in EndMT that still remain elusive and full understanding requires further deep exploration. Another limitation is that whether EndMT is a “cause” or an “effect” of CVD is not always clear and needs to be delineated in order to tease out a better prevention and/or treatment strategy for CVDs. EndMT can play a direct or indirect role in CVDs via releasing paracrine factors [174], but there are limited reports on the paracrine effect of EndMT in CVDs. Furthermore, EndMT is a context-dependent gradual transition of heterogeneous endothelial cells to mesenchymal cells. It becomes extremely important to dissect different CVD stages and their association with EndMT-stages to design EndMT-based treatment and/or prevention strategies. Other limitations are a lack of detailed characterization of different stages of EndMT and translational proof of concept in large animal models, which requires detailed systematic studies in large animal models. Nonetheless, several strategies to inhibit EndMT are being considered and deserve exploration in the context of CVDs.
